# LATS1 Is a Mediator of Melanogenesis in Response to Oxidative Stress and Regulator of Melanoma Growth

**DOI:** 10.3390/ijms22063108

**Published:** 2021-03-18

**Authors:** Urszula Kazimierczak, Ewelina Dondajewska, Maria Zajaczkowska, Marianna Karwacka, Tomasz Kolenda, Andrzej Mackiewicz

**Affiliations:** 1 Department of Cancer Immunology, Chair of Medical Biotechnology, Poznan University of Medical Sciences, Rokietnicka Street 8, 61-806 Poznan, Poland; ewelina.dondajewska@gmail.com (E.D.); mariazajaczkowska@gmail.com (M.Z.); majka.karwacka@gmail.com (M.K.); kolenda.tomek@gmail.com (T.K.); mackiewicz.aa@gmail.com (A.M.); 2 Department of Cancer Diagnostics and Immunology, Greater Poland Cancer Centre, Garbary Street 15, 61-866 Poznan, Poland

**Keywords:** melanoma, LATS1, melanogenesis, oxidative stress

## Abstract

The LATS1 kinase has been described as a tumor suppressor in various cancers. However, its role in melanoma has not been fully elucidated. There are several processes involved in tumorigenesis, including melanin production. Melanin content positively correlates with the level of reactive oxygen species (ROS) inside the cell. Accordingly, the purpose of the study was to assess the role of LATS1 in melanogenesis and oxidative stress and its influence on tumor growth. We have knocked down LATS1 in primary melanocytes and melanoma cells and found that its expression is crucial for melanin synthesis, ROS production, and oxidative stress response. We showed that LATS1 ablation significantly decreased the melanogenesis markers’ expression and melanin synthesis in melanocyte and melanoma cell lines. Moreover, silencing LATS1 resulted in enhanced oxidative stress. Reduced melanin content in LATS1 knocked down tumors was associated with increased tumor growth, pointing to melanin’s protective role in this process. The study demonstrated that LATS1 is highly engaged in melanogenesis and oxidative stress control and affects melanoma growth. Our results may find the implications in the diagnosis and treatment of pigmentation disorders, including melanoma.

## 1. Introduction

Hippo signaling is an evolutionarily conserved regulator of organ size and tumorigenesis. Activation of the pathway induces the interaction of a serine/threonine kinases cascade and adaptors, resulting in the phosphorylation of the effector molecules. In humans, these proteins include the serine/threonine kinases MST1/2 and LATS1/2, and the adaptor proteins MOB1 and SAV1. LATS1/2 kinases phosphorylate transcriptional co-activator paralogs YAP1 and TAZ to regulate transcription of proliferative and apoptotic factors. Non-phosphorylated YAP1/TAZ translocates to the nucleus and interacts with TEAD family of transcription factors. LATS1/2-phosphorylated YAP1/TAZ are rendered inactive via cytoplasmic sequestration. Inactivation of the MST and LATS kinases results in nuclear accumulation of YAP1/TAZ, and subsequent activation of their target genes, several of which are involved in cell proliferation. Hippo effectors YAP1 and TAZ contribute to multiple cancers’ metastatic behavior, including melanoma [1,2,3,4,5,6]. Although the role of Hippo pathway in melanoma has been proposed, no studies are describing its importance in melanogenesis. It is common that the melanin content affects melanoma progression and response to treatment [7,8]. Melanin biosynthesis is an oxidizing process that occurs in melanosomes inside the melanocytes. It involves the conversion of tyrosine to melanin in a set of enzymatic reactions catalyzed by tyrosinase (Tyr) and tyrosinase-related proteins 1 and 2 (TRP1, TRP2). The expression of Tyr, TRP1, and TRP2 is regulated by a microphthalmia-associated transcription factor (MITF) [9,10]. The presence of melanin in the skin has been considered as a double-edged sword: from one side, it protects melanocytes by absorbing UV radiation, but it also constitutes a source of high levels of intracellular reactive oxygen species (ROS) [11]. ROS are involved in the activation of melanin biosynthesis after the exposition of melanosomes to UVB radiation [12], which explains increased ROS levels in melanoma cells. Reactive oxygen species in low concentrations allow for proper intracellular signaling. They participate in the induction of immune defense against pathogens or cancer, e.g., by increasing the expression of suppressor genes such as *p53* and *PTEN*. On the other hand, their excessive accumulation in cells lead to oxidative stress (OS) and corresponds to the activation of transcription factors such as nuclear factor kappa-light-chain-enhancer of activated B cells (NF-κB), activator protein-1 (AP-1), hypoxia-inducible factor- 1α (HIF-1α) or signal transducer and activator of transcription 3 (STAT3), which activate genes involved in the inflammation, neoplastic conversion, proliferation and metastasis [13,14]. Therefore, ROS may favor both tumor suppression and progression, and it is crucial to regulate their levels to maintain homeostasis.

In this study, we report the involvement of the Hippo kinase LATS1 in the regulation of melanogenesis and oxidative stress responses. We also indicate the critical role of LATS1 in melanoma growth since both processes affect tumor formation. Up to our knowledge, this is the first report linking the Hippo signaling with melanogenesis control. Further analyses of the Hippo pathway in the context of melanin biosynthesis and oxidative stress will provide a better understanding of melanoma pathogenesis, which would help to develop more effective treatments.

## 2. Results

### 2.1. LATS1 Determines Melanin Production and Expression of Melanogenesis Markers in Primary Melanocytes and Melanoma Cells

**Figure 1:**
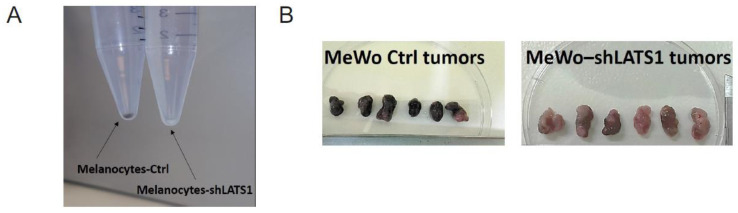

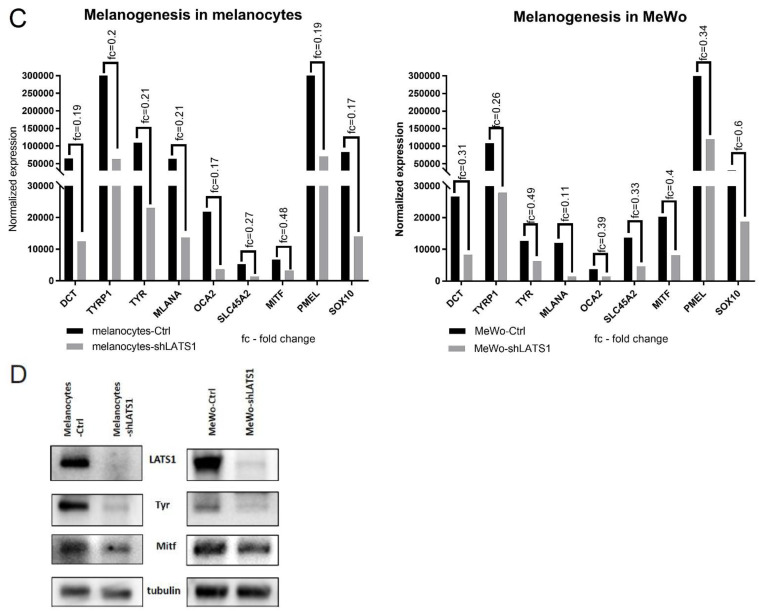
Melanin production and melanogenesis markers expression following LATS1 knockdown in primary melanocytes and MeWo cell line. (**A**) Pellets of cell-cultured with regular (left vial) or silenced (right vial) LATS1 expression. (**B**) Tumors formed in mice injected with MeWo cell line with LATS1 knockdown (bottom) vs. control (top). (**C**) Expression of genes involved in melanogenesis with fold changes of normalized expression values; black bars represent cells with regular LATS1 expression, grey bars represent cells with silenced LATS1. (**D**) Western blots of melanogenesis markers in cell lysates derived from control (Ctrl) and LATS1-knocked down (shLATS1) melanocytes and MeWo cells.

To address the importance of LATS1 in melanin biosynthesis, we knocked down LATS1 gene in highly pigmented primary human adult epidermal melanocytes (HEMa) as well as in MeWo melanoma cell line. We observed the pigmentary changes in time in vitro (melanocytes) and in vivo (MeWo) in nude mice. In both cases, the melanin content was visibly limited in cells lacking LATS1 (Figure 1A,B). The above changes were validated by mRNA sequencing and immunoblotting of cell lysates. We observed a significant decrease in all tested melanogenesis markers, including tyrosinase and MITF, both on a transcript (Figure 1C), and protein (Figure 1D) levels.

### 2.2. LATS1 Regulates the Proliferation of Primary Melanocytes and Determines Tumor Growth Kinetics

**Figure 2:**
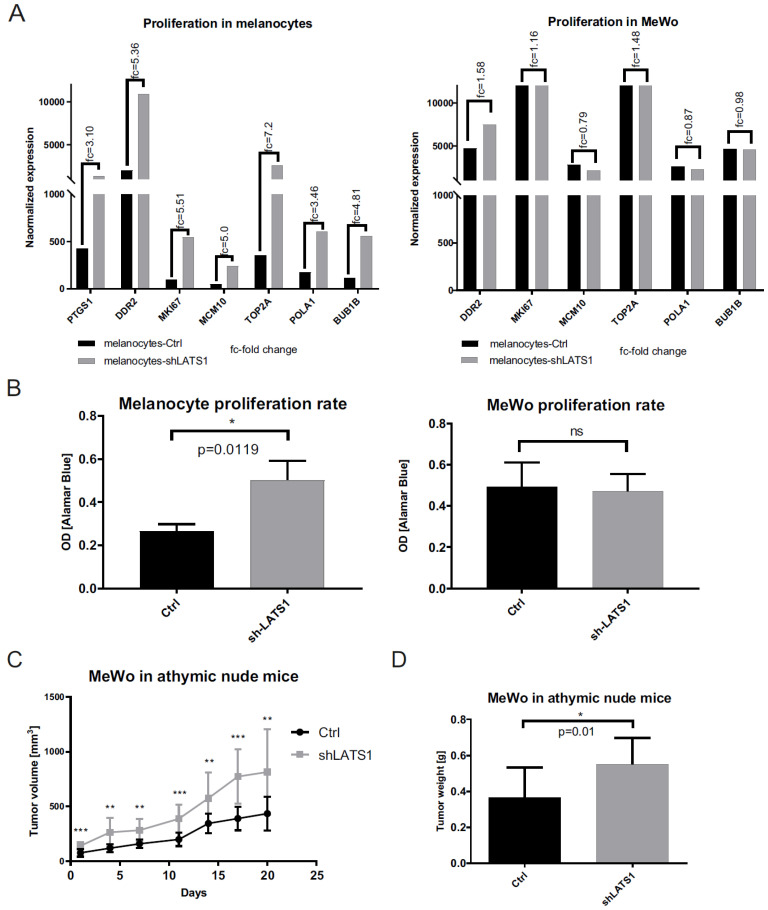
Proliferation markers and proliferation rates in primary melanocytes and MeWo cell line in vitro and in vivo following LATS1 silencing. (**A**) mRNA expression analysis of proliferation markers in primary melanocytes (left) and MeWo cells (right) before (black bars) and after (grey bars) LATS1 knockdown, with fold changes of normalized expression values. (**B**) Proliferation rates of primary melanocytes (left) and MeWo cells (right) either LATS1 depleted (grey bars) or non-modified cells (black bars) measured with functional metabolic Alamar Blue proliferation assay. (**C**) Growth kinetics of MeWo tumors with LATS1 knockdown (grey curve) vs. control (black curve). (**D**) Volumes of MeWo tumors with regular (black bars) and knocked down LATS1 (grey bars); * *p* ≤ 0.05, ** *p* ≤ 0.01, *** *p* ≤ 0.001, ns—not significant.

Following the demonstration that LATS1 is crucial for melanogenesis, its effects on other functions of melanocytes and melanoma cells were analyzed. First, the influence of LATS1 knockdown on proliferation was studied. For in vitro proliferation, the matabolic resazurin reduction assay was used and RNAseq to analyze the expression of proliferation markers. A significant increase in proliferation rates (Figure 2B) and the expression level of all the proliferation-related genes tested following LATS1 knockdown (Figure 2A) were observed. However, only in the case of melanocytes LATS1 silencing did not affect the proliferation of the melanoma cells in vitro (Figure 2A,B), but it significantly increased tumor growth kinetics, and tumor weight after xenografting them into nude mice (Figure 2C,D).

### 2.3. LATS1 Knockdown Enhances Reactive Oxygen Species Production in Primary Melanocytes and Melanoma Cells

**Figure 3:**
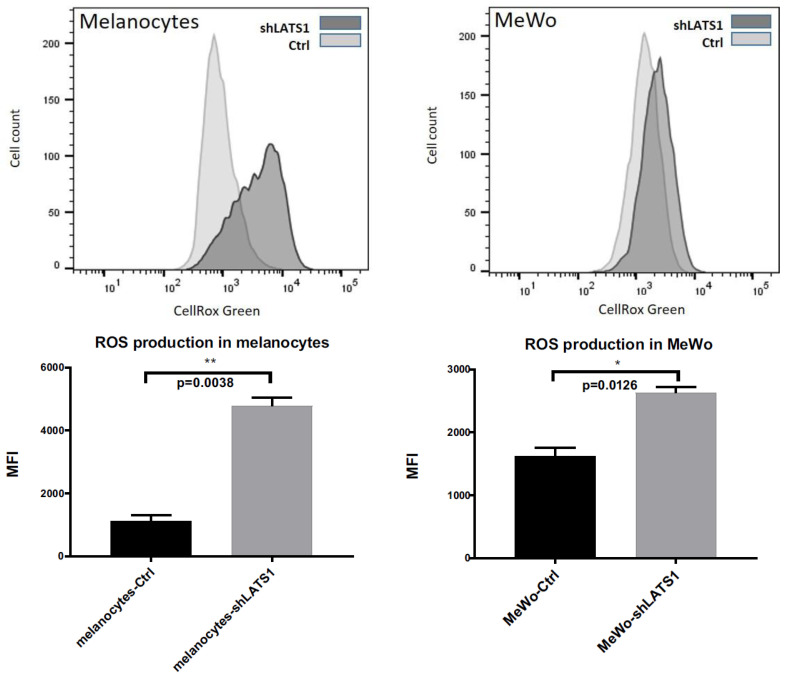
Intracellular ROS levels following LATS1 knockdown. Flow cytometry analyses of the levels of reactive oxygen species (ROS) in primary human melanocytes (left) and MeWo cells (right); CellRox Green regent was used as ROS sensor with green fluorescence enhanced by oxidation; mean fluorescence intensity (MFI) was used as a measure of ROS content inside the cells; * *p* ≤ 0.05, ** *p* ≤ 0.01.

Since melanin is considered a reactive oxygen species (ROS) scavenger, LATS1 involvement in response to oxidative stress was tested. The cytometric measurement of intracellular ROS demonstrated that in both melanocytes and melanoma cells in vitro, LATS1 knockdown was associated with a significant increase in ROS levels (Figure 3).

### 2.4. LATS1 Regulates Oxidative Stress-Responsive and Cellular Stress-Related Genes Expression in Melanocytes but Not in Melanoma Cells

**Figure 4:**
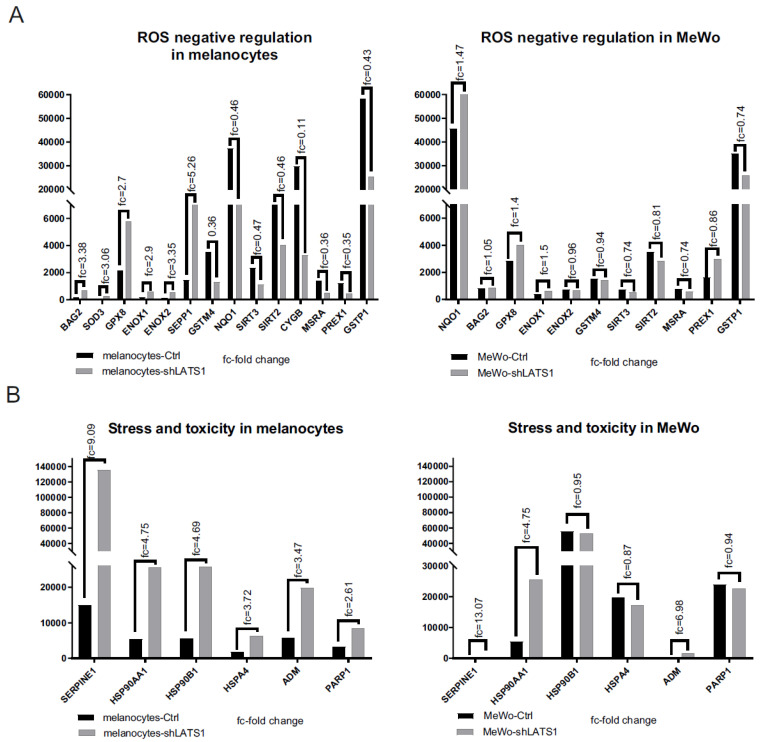
Expression of oxidative stress and toxicity markers following LATS1 knockdown. Comparison of the expression of the genes negatively regulating oxidative stress (**A**) and stress and toxicity markers (**B**) in primary melanocytes (left) and MeWo cells (right), with fold changes of normalized expression values. black bars represent cells with regular LATS1 expression; grey bars represent cells with LATS1 knocked down.

Following the demonstration that LATS1 highly affects ROS production, the expression levels of ROS-responsive genes and the expression of the genes related to cellular stress and toxicity were studied. Most of the genes, which negatively regulate oxidative stress, displayed lower expression rates following LATS1 knockdown, in contrast to stress-related genes, which expression was remarkably increased (Figure 4A,B). However, those results concerned melanocytes only. In contrast, in MeWo cells LATS1 level was not associated with the expression of genes analyzed.

### 2.5. LATS1 Regulates the Expression of Hypoxia-Related Genes

**Figure 5:**
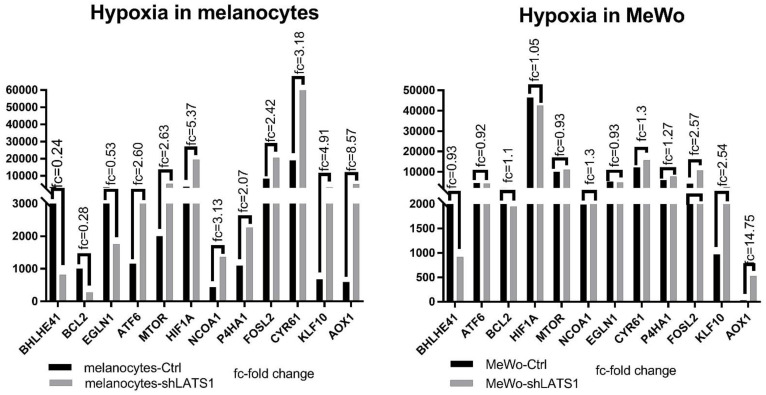
Expression of hypoxia-related genes in LATS1 knocked down cells. Comparison of the expression of the genes regulated under hypoxia in primary melanocytes (left) and MeWo cells (right), with fold changes of normalized expression values; black bars represent cells with regular LATS1 expression, grey bars represent cells with LATS1 knocked down.

Since oxidative stress is strictly related to hypoxia, we assessed the role of LATS1 in the regulation of hypoxia-related genes. We found that LATS1 affects the expression of most genes studied in melanocytes but not in MeWo cells. However, in MeWo cells, a significant increase of expression in one of the crucial genes involved in hypoxia and oxidative stress regulation- aldehyde oxidase (AOX1) was seen (Figure 5).

### 2.6. LATS1 Silencing Drives Minor Changes in YAP1/TAZ Expression in Primary Melanocytes but Not in MeWo Cells

**Figure 6:**
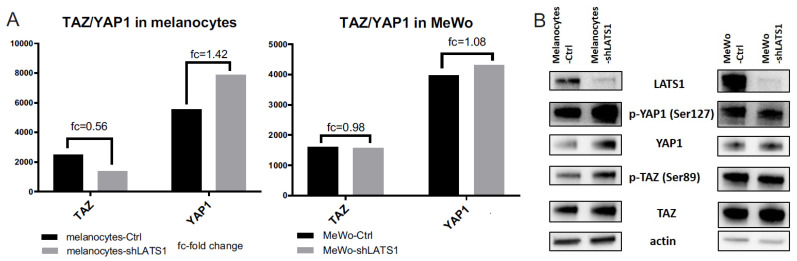
YAP1/TAZ expression in LATS1 knocked down cells. (**A**) Expression of YAP1 and TAZ genes in primary melanocytes (left) and MeWo cells (right); black bars—cells with regular LATS1 expression, grey bars—cells with LATS1 knocked down. (**B**) Western blotted lysates of control (Ctrl) and LATS1 knocked down (shLATS1) melanocytes (left panel) and MeWo (right panel).

Since LATS1 is a master regulator of Hippo signaling targets YAP1 and TAZ, the expression of both genes and their protein products was tested. There were only slight differences in YAP1 and TAZ transcripts expression in melanocytes but not in MeWo cells (Figure 6A). Similarly, only in HEMa cells, the protein levels’ differences were observed: in this case, both YAP1 and its phosphorylated forms were slightly elevated after LATS1 knockdown (Figure 6B).

## 3. Discussion

In this study, we discovered a new function of the central kinase of Hippo signaling- LATS1. We demonstrated that LATS1 is highly involved in melanin biosynthesis as well as in tumor growth control. Then, we linked those phenomena with an oxidative stress response. Our work showed that LATS1 ablation significantly impaired melanogenesis and that the cells with the reduced melanin content proliferated faster in vitro and formed larger tumors in mice. Moreover, knocking down LATS1 increased hypoxia state and oxidative stress inside the melanocytes and melanoma cells. Our findings indicate that LATS1 regulates melanogenesis by modulating ROS content within the cell. That emphasizes the possible mechanism of melanotic melanoma development.

So far, the role of melanin in melanoma has not been fully understood. Some reports demonstrate that melanin causes higher oxygen consumption and aerobic glycolysis stimulation, providing energy to the tumor [15]. However, there are also studies showing that the presence of melanin inhibits melanoma metastasis [7]. Since melanin biosynthesis affects melanoma cell behavior, pigmentation appears to be an essential factor for melanoma metastasis and bears potential clinical implications for melanoma diagnosis and treatment. We showed that tumors lacking the pigment displayed significantly higher growth kinetics than melanotic ones, thus concluded that melanin biosynthesis decreases tumor growth. Inhibition of melanogenesis may be associated with activation of JAK2-STAT6 signaling [16]. IL-6 suppresses the expression of the tyrosinase and MITF [17], TNF-α reduces the half-life of tyrosinase [18]. Additionally, IL-4 may downregulate the expression of melanogenesis associated genes [19]. Any impairment that decreases melanin biosynthesis affects either melanocyte number or their function [20].

We found that both melanogenesis and melanoma progression were highly associated with the Hippo pathway. LATS1 knockdown suppressed the expression of pigmentation related genes, including tyrosinase, TYRP1/2, and OA1. LATS1 ablation also downregulated MITF, which is considered a master gene in melanocyte differentiation. MITF directly influences the expression of a diverse set of melanocyte genes, as well as a wide range of genes engaged in multiple other processes such as cell cycle regulators (CDK2, TBX2, p21, p16/Ink4a), survival and metastasis drivers (BCL2,c-Met), miRNA processing (Dicer), cAMPlevels (PDE4D3), or melanogenesis markers mentioned above. MITF expression is driven by several transcription factors, including Sox10, Pax3, CREB, and the Wnt pathway [21]. Out of those factors, Sox10 is an essential developmental regulator of melanogenesis. In the absence of Sox10, MITF cannot induce the expression of tyrosinase or *TYRP2* [21,22,23,24]. Sox10 mutations most commonly result in Waardenburg-Shah syndrome and Hirschsprung disease, both manifesting with pigmentary abnormalities [25]. Moreover, in melanoma cells, Sox10 ablation suppresses melanoma progression [21]. Our results showed a significant decrease in Sox10 expression after LATS1 knockdown, which confirms its essential role in melanogenesis. It is possible that LATS1 regulates MITF expression through regulating i.a. the expression of Sox10. Deregulated MITF is implicated in the pathogenesis of several tumors [26]. Most of all, much of the pathophysiology of melanoma is driven by the activity of MITF [27]. It has been demonstrated that the MITF-low population has exacerbated tumorigenic properties. However, the MITF-high population grew faster but displayed poor tumorigenicity when injected in mice [28]. Cheli et al. showed that MITF silencing increased the invasive and metastatic properties of melanoma cells. In that case, inhibition of MITF might be deleterious in melanoma treatment [29]. Our studies suggest that downregulation of MITF may constitute one of the critical molecular events that drive melanoma growth.

It has been reported that MITF expression and its target expression are downregulated under hypoxic conditions leading to the dedifferentiation of melanoma cells, which become more aggressive [29,30]. There are two distinct mechanisms to generate a hypoxia gene signature: one is dictated by low oxygen in the microenvironment and the other by the cell’s intrinsic state [31]. In our studies, the cells with LATS1 knockdown seem to exhibit a constitutive hypoxia signature, even though grown under normoxic conditions. We observed that a high hypoxia gene expression signature is associated with low levels of MITF mRNA. Since these cells are cultured in normoxic conditions, the lack of correlation between MITF and the hypoxia response cannot arise due to low oxygen, but seems to be attributed to LATS1 knockdown. Several studies have examined the role of hypoxia in melanoma. It has been demonstrated that under hypoxia, MITF is transcriptionally downregulated via an indirect mechanism involving hypoxia-inducible factor 1 (HIF1)-mediated upregulation of the transcription factor bHLHE40/DEC1 [26]. HIF1 is considered as a target of MITF but also its regulator [32]. In our model HIF1 expression is stable in melanoma cell line and rises after LATS1 knockdown, but increases in modified melanocytes. Still, in both cases, MITF expression remains downregulated, which indicates that most probably this is an effect of LATS1 silencing.

Interestingly, HIF1α is also considered a key regulator of transcriptional responses to oxidative stress [33]. It has been demonstrated that the mitochondrial ROS may stabilize HIF1α, which further promotes cell invasion and vasculogenic tumor mimicry [34]. The elevated ROS production, which we observed in our studies, was linked to an elevated hypoxia state inside the cell raised after LATS1 knockdown. These data indicate that the Hippo pathway, particularly LATS1 kinase, may regulate hypoxia by modulating oxidative stress.

ROS generation impacts cell signaling and homeostasis under both physiological and pathophysiological conditions [35]. Excessive ROS production leads to oxidative stress (OS), which is a consequence of the imbalance between oxidants and antioxidants in favor of the oxidants, potentially leading to biological damage [36]. ROS may be generated due to increased metabolism of transformed cells, immune reactions, altered antioxidant system, UV radiation, and melanin production [37]. Compared to cells without pigment, melanocytes show an increased level of ROS, due to melanin content. Melanin protects cells against the harmful effects of UV, but at the same time, mediates oxidation reactions [38]. The excessive accumulation of ROS in melanocytes leads to neoplastic transformation [39]. Disruption of melanosomal melanin may increase oxidative stress and lead to melanoma progression [40].

The role of ROS in cancer remains elusive. ROS have emerged as mediators of signaling pathways engaged in cell proliferation, tumor initiation, and promotion. Thus, they play essential roles in almost every stage of melanoma development, from cell proliferation, DNA damage to invasion and drug resistance [41,42]. Depending on their concentration, ROS can act as tumor promoting and tumor suppressing agents to keep intracellular homeostasis. ROS may induce cancer cell proliferation, evasion of cell death pathways, angiogenesis, and metastasis [43]. Several studies have shown that the generation of large amounts of ROS enhances the aggressiveness of tumors. Paradoxically, oxidative stress can also be toxic and induce cell death, depending on the redox imbalance’s duration and extent [44,45]. By upregulating ROS responsive genes’ expression, cancer cells have developed a defense mechanism to prevent lethal ROS accumulation. We observed a similar mechanism in primary melanocytes: the elevated expression of the antioxidant system most probably equilibrated significantly enhanced oxidative stress to prevent cell death. Similarly, in the melanoma cell line LATS1 silencing caused an enhanced generation of ROS. However, the level of ROS was not only accurate to keep the cells alive but also it was high enough to promote tumor growth in mice.

One of the most important factors influencing both oxidative stress and hypoxia is aldehyde oxidase (AOX1), which produces hydrogen peroxide and, under certain conditions, it can catalyze superoxide formation. The relationship between AOX1 and cancer is ambiguous, depending on tumor type. It has been reported that AOX1 inhibited the development of breast cancer [46], but at the same time, it could promote prostate and rectal cancers [47]. AOX1 enhances the proliferation and invasion and inhibits apoptosis via ROS production, but it also upregulates the CD133 and PI3K/Akt pathway to promote disease progression [48]. We observed highly elevated AOX1 gene expression in LATS1 knocked down cells, both in melanocytes and melanoma cell line.

Moreover, in both cell types, endogenous ROS levels were increased and Akt phosphorylation (data not shown). Those results are consistent with the hypothesis that AOX1 promotes cancer; however, the mechanism is much more complicated, taking into account the Hippo pathway’s role in this process. The hypothesis is that the downregulation of LATS1 enhances ROS production, promoting an intrinsic hypoxia state with elevated AOX1 expression, which upregulates cancer stem cell marker CD133 resulting in enhanced tumor growth. However, both hypoxia and ROS mediated effects involve complex interactions between several different signaling pathways; thus, further research needs to confirm that hypothesis.

The major finding of our study is that LATS1 silencing leads to a significant decrease in the expression of melanogenesis driver genes and melanin content, both in primary melanocytes and melanoma cells. Accordingly, we suggest that the Hippo pathway is a strong regulator of melanogenesis, and through a molecular cascade involving MITF and oxidative stress response, controls melanoma growth. Still, some issues remain open. Most of all, what is the role of the direct LATS1 targets- YAP1 and TAZ in between those events? Those genes were expressed in both cell lines without significant changes before and after LATS1 knockdown on the transcriptional levels. There were still no significant differences between YAP1 and TAZ and their phosphorylated versions on the protein level. We have observed only a slight increase in YAP1 and phospho-YAP1 levels after LATS1 knockdown in primary melanocytes. That leads us to conclude, that unlike in other models, LATS1 is not a primary kinase for YAP1 inactivating phosphorylation in melanocytes and melanoma. This issue will be explored in our next study.

It is also noteworthy that although LATS1 silencing results in decreased expression of the genes engaged in melanogenesis and increased ROS production both in primary human adult epidermal melanocytes and MeWo cell line, it exerts different effects on the other characteristics. Those differences most likely result from the different origins of both cell types and their genetic backgrounds. Melanocytes represent highly pigmented normal skin tissue, while MeWo constitutes the established cell line originating from the lymph node metastasis of melanoma and shows no pigmentation in vitro. Although both cell lines are wild type regarding BRAF, MeWo cells have mutations in several other genes, eg. *p53*, *MAPK3*, *CDKN2A*, which undoubtedly influence certain cellular processes. That is an important matter to consider for further understanding the role of LATS1 in melanogenesis.

To summarize, this paper reports that regulation of human skin pigmentation and melanomagenesis are highly dependent on LATS1 kinase and oxidative stress in response to LATS1 ablation. The study provides valuable knowledge about signaling pathways engaged in melanogenesis and melanoma pathophysiology, which may help select targets for better diagnosis and treatment.

## 4. Materials and Methods

### 4.1. Cell Culture and Reagents

The primary human adult epidermal melanocytes (HEMa) were purchased from ATCC (Manassas, VA, USA) and cultured in Derman Cell Basal Medium (ATCC) supplemented with Adult Melanocyte Growth Kit (ATCC) according to the manufacturer’s recommendations. The human melanoma MeWo cell line was obtained from the Jagiellonian University (Krakow, Poland) and was maintained in Dulbecco’s Modified Eagle’s Medium (DMEM; Biowest, Nuaille, France) supplemented with 10% fetal bovine serum (FBS, Biowest) and antibiotics (penicillin/streptomycin, 100 units/mL, Biowest). Following transduction, media were supplemented with 2 or 3 µg/mL of puromycin (Sigma-Aldrich, Munich, Germany), respectively. Alamar Blue (Bio-Rad Laboratories, Hercules, CA, USA) was used to measure cell proliferation rates. Shortly, cells were plated onto 96-well plates in a number of 10^4^ cells per well, and cultured for 72 h; 1/10 volume of Alamar Blue was added to each well and after the reaction was developed, assays were measured spectrophotometrically to determine the optical density (OD) values.

### 4.2. Lentivirus Production and Transduction

Lentiviral vectors were produced in 293 T human embryonic kidney cells using a second-generation packaging system, as published previously [49]. Shortly, 3 µg pMD2G, 7 µg pPAX, and 10 µg pLKO.1-shGFP or pLKO.1-shLATS1 per 100 mm culture plate were used for co-transfections using the calcium phosphate precipitation method. The shRNA hairpins against GFP and LATS1 were previously described [50]. Viral supernatants were collected 48 h post-transfection, concentrated on Amicon Ultra centrifugal filter units Ultra-50, MWCO 100 kDa, aliquoted and stored in −80 °C. Transductions were conducted on 6-well culture dishes using 1/20 volume of the concentrated virus. Forty-eight hours post-transduction cells were subjected for selection in 2 or 3 µg/mL of puromycin for HEMa or MeWo cell line, respectively. The transduced cells were further cultured in the presence of puromycin.

### 4.3. RNA Sequencing

Total RNA isolation and mRNA sequencing and were performed by CeGat GmbH (Tubingen, Germany). The library from each sample was prepared for sequencing using the Illumina TruSeq Stranded mRNA Library Preparation Kit. 100 ng of RNA was used to prepare the RNA libraries. The libraries were sequenced on Illumina NovaSeq 6000, 2 × 100 bp. Depths of 30 million paired-end 100  bp reads were generated for each sample. Demultiplexing of the sequencing reads was performed with Illumina bcl2fastq. Adapters were trimmed with Skewer (version 0.2.2). Quality trimming of the reads has not been performed. RNA-Seq trimmed raw reads were aligned to the human reference genome (hg19) using STAR. Differential expression analysis between groups was performed with DESeq2 in R. DESeq2 uses a negative binomial generalized linear model to test for differential expression based on gene counts.

### 4.4. Protein Extraction and Western Blot

Cells were washed twice with phosphate-buffered saline (PBS; Biowest), and collected into fresh tubes using cell scrapers. Proteins were extracted in ice-cold 1× RIPA lysis buffer (Sigma-Aldrich, Munich, Germany) containing 10× diluted protease inhibitor cocktail (Sigma Aldrich). Extracts were kept on ice for 20 min and centrifuged at 13,000× *g* RPM for 15 min at 4 °C. Supernatants were stored at −80 °C. Proteins were quantified by BCA assay (Thermo Fisher Scientific, Waltham, MA, USA). For immunoblotting, 15 μg of whole protein extracts were loaded onto 4-20% gradient polyacrylamide gels (Bio-Rad Laboratories, Hercules, CA, USA) and separated by SDS-PAGE. Proteins were then electroblotted onto PVDF membranes (Bio-Rad Laboratories) using Trans-Blot Turbo Transfer System (Bio-Rad Laboratories). Unspecific binding sites were blocked using 5% BSA resolved in tris buffered saline containing 0,1% Tween 20 (TBS-T) (further referred to as blocking buffer). Primary anti-human monoclonal antibodies specific to LATS1, phosphorylated TAZ (Ser89), TAZ, phosphorylated YAP1 (Ser127), YAP1 (CST), MITF, tyrosinase (Thermo Fisher Scientific), were diluted 1:1000 in blocking buffer and incubated overnight at 4 °C with the respective fragments of the membrane. After washing, the membranes were incubated with anti-rabbit (for CST antibodies) or anti-mouse (for Thermo Fisher Scientific antibodies) IgG conjugated to horseradish peroxidase (HRP) (CST), diluted 1:2000 in blocking buffer. All washing steps were performed using TBS-T buffer. Proteins were visualized by chemiluminescence using WesternBright Quantum HRP substrate (Advansta, Menlo Park, CA, USA) and CCD imager (G:BOX; Syngene, Cambridge, UK).

### 4.5. Oxidative Stress Detection

The intracellular level of reactive oxygen species was measured using CellRox Green Reagent (Thermo Fisher Scientific, Waltham, MA, USA), according to the manufacturer’s instructions. One day prior the experiment cells were plated onto 6-well culture plates. When they reached 60–70% confluency, 5 µM CellRox Reagent was added to each well for 30 min at 37 °C. Following incubation cells were trypsinized, collected and washed twice in PBS, and the mean fluorescence intensity (MFI) for each sample was measured using FACSAria flow cytometer (BD, Franklin Lakes, NJ, USA). The results were analyzed with FlowJo software (Tree Star Inc, Ashland, OR, USA).

### 4.6. Mice

All in vivo experiments were approved by the Local Ethics Committee in Poznan (LKE; protocol number 49/2016 from August 2016). 8–9 weeks old athymic nude Crl:NU(Ncr)-Foxn1nu female mice (Charles River Laboratories) were purchased from AnimaLab (Poznan, Poland). Tumor cells (MeWo) with LATS1 knocked down or mock vector treated were cultured as described above. When reached 70–80% confluency, they were collected, washed twice with PBS and resuspended in fresh PBS, 2 × 10^7^/mL. Just prior the injection, cells were mixed 1:1 with the growth factor reduced matrigel (BD Biosciences) and 200 µL of cell suspension was injected subcutaneously (s.c.) in both flanks of the body using 1-mL syringes and insuline needles. Each group contained 6 mice/12 tumors. Tumors were measured every 2–3 days. Once at least one of the tumor xenografts in a group reached volume >1000 mm^3^, mice were sacrificed using a carbon dioxide euthanasia system. Tumors were excised, photographed, and tumor tissue was stored in the form of lysates for further analysis.

### 4.7. Statistical Analyses

The Shapiro–Wilk normality test, t-test, and Mann–Whitney U test were used to analyze the differences between groups of data tested; *p* < 0.05 was used to determine statistical significance. All statistical analyses were performed using GraphPad Prism 7 (GraphPad, San Diego, CA, USA).

## Data Availability

The data presented in this study are available on request from the corresponding author. The data are not publicly available due to property rights.
